# Does family communication moderate the association between adverse childhood experiences and emotional and behavioural problems?

**DOI:** 10.1186/s12889-020-09350-9

**Published:** 2020-08-20

**Authors:** Miriama Lackova Rebicova, Zuzana Dankulincova Veselska, Daniela Husarova, Daniel Klein, Andrea Madarasova Geckova, Jitse P. van Dijk, Sijmen A. Reijneveld

**Affiliations:** 1 Department of Health Psychology and Research Metodology, Medical Faculty, PJ Safarik University, Trieda SNP 1, 040 01 Kosice, Slovak Republic; 2 Graduate School Kosice Institute for Society and Health, PJ Safarik University, Trieda SNP 1, 04001 Kosice, Slovak Republic; 3 Institute of Mathematics, Faculty of Natural Sciences, P.J. Safarik University, Srobarova 2, 04154 Kosice, Slovak Republic; 4 Olomouc University Social Health Institute, Palacky University, Univerzitni 22, 771 11 Olomouc, Czech Republic; 5 Department of Community and Occupational Medicine, University Medical Center Groningen, University of Groningen, Antonius Deusinglaan 1, 9713 AV Groningen, the Netherlands

**Keywords:** Family communication, Adverse childhood experiences, Emotional problems, Behavioural problems, Adolescents

## Abstract

**Background:**

Adverse childhood experiences (ACEs) and poor family support and communication can increase emotional and behavioural problems (EBP). Therefore, we assessed the association of difficult communication with mother and with father separately with both emotional and behavioural problems (EBP), and whether adolescents’ communication with mother and with father moderates the association of adverse childhood experiences (ACE) with the EBP of adolescents.

**Methods:**

We used data from the Health Behaviour in School-aged Children study conducted in 2018 in Slovakia, comprising 5202 adolescents aged from 11 to 15 (mean age 13.53; 49.3% boys). EBP were measured using the Strengths and Difficulties Questionnaire. We used generalized linear regression adjusted for age, gender and family affluence to explore the modification of the associations between ACE and EBP by communication (easy vs. difficult communication) with mother and father.

**Results:**

Difficult communication or a complete lack of communication due to the absence of mother and father increased the probability of emotional (exp (b): 0.96, 95% CI: 0.92|1.00; and 0.95, 95% CI: 0.91|0.99, respectively) and also of behavioural problems (exp (b): 0.96, 95% CI: 0.92|1.00; and 0.94, 95% CI: 0.90|0.97, respectively). We found a statistically significant interaction of communication with father on the association of ACE with EBP, showing that the joint effects were less than multiplicative.

**Conclusion:**

Difficult communication with mother and father is related to EBP among adolescents, and adolescents’ communication with father moderates the association of ACE with both emotional and behavioural problems among adolescents.

## Background

Adverse childhood experiences (ACE) regard a wide range of negative events that occur at a young age, such as abuse and/or neglect towards a child, domestic violence towards a youth’s mother, household substance abuse, household mental illness, parental separation/divorce and other events [1, 2]. Experiences of long-term ACE can cause serious emotional and behavioural problems (EBP) throughout the life of a child [1, 3–5]. In addition, ACE has been shown to have a dose–response relationship with various mental health conditions, including depression, anxiety, panic reactions, hallucinations, psychoses and suicide attempts [6–14]. The mechanisms for these associations may involve the modified physiological development of children due to experienced chronic stress [15, 16] or the adoption of behaviours that harm their physical and mental health [3, 17].

Family factors, e.g. family support and family communication, are associated with positive adolescent development, i.e. are protective factors [18, 19]. Worsening or even a complete lack of family support and communication was found to be associated with a decline in adolescent mental health, which points to the importance of family communication with both parents [20–22] and to the nature of family relationships in adolescent health [22, 23]. Worse child-parent family relationships were associated with anxiety and depression [21, 23–26]. Even more, in relationships between adolescents and parents with insufficient or lacking communication, adolescents were found to be at increased risk of mental health problems [21, 25–28]. Most of the available evidence has focused on the role of overall communication with parents on adolescents’ mental health regardless of the gender of the parent [18, 19, 21, 22]. Moreover, no attention has been paid to the potential role of communication with mother and with father as moderators of the relationship between ACE and EBP.

Low socioeconomic position may be considered as one of the ACE itself and causes early life stress [1, 27, 28]. More experience with financial stress was found to be associated with poorer mental health [29], and also to be related to more behavioural problems [1, 30].

The aim of this study is to assess the association of difficult communication with mother and with father separately with both emotional and behavioural problems, and whether adolescents’ communication with mother and with father moderates the association of ACE with both emotional and behavioural problems among adolescents.

## Methods

### Sample and procedure

We used data from the Health Behaviour in School-aged Children (HBSC) study conducted in 2018 in Slovakia. More detailed information about two-step sampling process and procedure used to acquire this nationally representative sample could be found in our previously published paper [1, 31]. In the first step, 140 larger and smaller elementary schools located in rural as well as urban areas from all regions of Slovakia were asked to participate. These were randomly selected from a list of all eligible schools in Slovakia obtained from the Slovak Institute of Information and Prognosis for Education. The school response rate (RR) was 77.9%. In the second step, we obtained data from 8405 adolescents from the fifth to ninth grades of the elementary schools in Slovakia in the target group of 11 to 15 years old (mean age 13.43; 50.9% boys); one class per grade was selected. Respondents with missing responses were excluded (3203), leading to a final sample of 5202 adolescents (mean age 13.53; 49.3% boys).

The study was approved by the Ethics Committee of the Medical Faculty at the P.J. Safarik University in Kosice (16 N/2017). Parents were informed about the study via the school administration and could opt out if they disagreed with their child’s participation. Then, written consent from study participants was obtained. Participation in the study was fully voluntary and anonymous, with no explicit incentives provided for participation.

### Measures

*Emotional and behavioural problems (EBP)* were measured using the Strengths and Difficulties Questionnaire (SDQ), which includes 25 items on psychological attributes [32], of which we used the 20 problem items. Response categories were: not true (0), somewhat true (1), certainly true (2). Detailed information about compute of emotional (internalizing) and behavioural (externalizing) problems could be found in our previously published paper [1, 33]. Cronbach’s alpha in our sample was 0.73 for the whole scale, and 0.71 for the internalizing and externalizing subscales, respectively.

*Adverse childhood experiences (ACE)* were measured using a series of questions on events: “Have you ever experienced any of the following serious events? (Death of a brother/sister, Death of your father/mother, Death of somebody else you love, Long or serious illness of yourself, Long or serious illness of one of your parents or of someone else close to you, Problems of one of your parents with alcohol or drugs, Repeated serious conflicts or physical fights between your parents, Separation/divorce of your parents, Separation of your parents due to work abroad, Moving to another house/flat, or city/village, Transfer to another school). Questions about childhood abuse and neglect were not included in the ACE questionnaire measured for this study. The response categories were “yes” and “no”. More detailed information about creating a sum score of ACE experienced could be found in our previously published paper [1]. Based on the literature and the distribution of categories, we next categorised the number of ACE into three categories: no ACE, one or two ACE and three or more ACE [1, 34–36].

*Communication with parents/stepparents* was measured with the question “How easy is it for you to talk to the following persons about things that really bother you? (mother, father, stepmother, stepfather)”. The response categories were very easy / easy / difficult / very difficult / don’t see or have this person. Questions about communication with mother and stepmother were merged, as were questions about communication with father and stepfather. Both combined variables were dichotomized into two categories – easy / difficult or don’t see or have this person. We combined these two categories (difficult communication / don’t have this person), because in both situations the parent does not fulfil his/her role.

*Socioeconomic position* was measured using the Family Affluence Scale III (FAS-III), which consists of six questions: “Does your family own a car, van or truck?” (No / Yes, one / Yes, two or more), “Do you have your own bedroom for yourself?” (Yes / No), “How many computers does your family own?” (None / One / Two / More than two), “How many bathrooms (room with a bath/shower or both) are in your home?” (None / One / Two / More than two), “Does your family have a dishwasher at home?” (Yes / No), “How many times did you and your family travel out of your country for a holiday/vacation last year?” (Not at all / Once / Twice / More than twice). We computed sum score, which we converted into a ridit score ranging from 0 to 1. We then created tertile categories of low (0 to 0.333), medium (0.334 to 0.666) and high (0.667 to 1) socioeconomic position [31, 37].

### Statistical analyses

First, we described the sample using descriptive statistics in Table 1. Second, we assessed the association of communication with mother and with father with emotional problems and behavioural problems (Model 1). Third, we assessed the associations of the number of ACE and of communication with mother and with father with emotional problems and behavioural problems (Model 2). Finally, we explored the modification of the associations of ACE with emotional problems and behavioural problems by communication with mother and with father separately (Model 3). For these three steps we used generalized linear models adjusted for age, gender and family affluence. To overcome the problem of normality assumption of the dependent variable, the gamma distribution with log link function was used. Because of the log transformation of the dependent variable, the regression coefficients were transformed exponentially and displayed as such in Table 2 to show the effect of the independent variables on the non-transformed dependent variable. Statistical analyses were performed using SPSS v.20.

## Results

### Background characteristics

The background characteristics of the sample are presented in Table 1. We found that 15.2% of the study sample reported no ACE, 46.2% reported 1–2 ACE and 38.6% 3 or more ACE (Table 1). Of the respondents, 79.3% reported easy communication with mother/step-mother and 67.7% easy communication with father/step-father.

**Table 1:** Descriptive statistics of the sample (HBSC-study, Slovakia 2018, 11–15 years old, *N* = 5202)

	Total
	***N*** = 5202
**Gender (*N*, %)**	
Boys	2562 (49.3)
**Age (mean, SD)**	13.53 1.31
**FAS (*N*, %)**	
Low	1466 (28.2)
Middle	1508 (29.0)
High	2029 (39.0)
**ACE (*N*, %)**	
No ACE	790 (15.2)
1–2 ACE	2402 (46.2)
3 and more ACE	2010 (38.6)
**Communication with mother/stepmother (*N*, %)**	
Easy	4092 (79.3)
**Communication with father/stepfather (*N*, %)**	
Easy	3514 (67.7)
**Emotional and behavioural problems (mean, SD)**	
Emotional problems	15.13 3.34
Behavioural problems	16.48 3.28

*HBSC-study* Health Behaviour in School-Aged Children study, *N* number of respondents, *FAS* Family affluence, *ACE* adverse childhood experiences, *SD* standard deviation

Note. Only valid percentages are presented; missing values: FAS = 119 (3.8%), communication with mother = 42 (0.8%), communication with father = 11(0.2%)

**Table 2:** The association between communication with mother and with father and EBP, and the moderation of the associations between ACE and EBP by communication with mother and with father from generalized linear models adjusted for age, gender and FAS (exp (b) 95% Confidence intervals) (Slovakia 2018, 11–15 years old, *N* = 5202)

	Emotional problems			Behavioural problems		
	Model 1	Model 2	Model 3	Model 1	Model 2	Model 3
***Mother***						
**ACE**						
0		Ref.	Ref.		Ref.	Ref.
1–2		1.03 (1.01|1.04)**	1.03 (1.01|1.05)**		1.02 (1.00|1.04)*	1.02 (1.00|1.04)*
3 and more		1.08 (1.06|1.11)***	1.09 (1.07|1.12)***		1.08 (1.06|1.09)***	1.08 (1.07|1.11)***
**Communication with mother**						
easy	Ref.	Ref.	Ref.	Ref.	Ref.	Ref.
difficult	0.11 (0.10|0.13)***	1.12 (1.11|1.13)***	1.14 (1.09|1.19)***	0.10 (0.09|0.12)***	1.11 (1.09|1.12)***	1.13 (1.08|1.16)***
ACE*Communication with mother			ns			ns
1–2 ACE*difficult			0.98 (0.93|1.02)			0.98 (0.94|1.03)
3 ≥ ACE*difficult			0.98 (0.93|1.02)			0.97 (0.93|1.01)
***Father***						
**ACE**						
0		Ref.	Ref.		Ref.	Ref.
1–2		1.02 (1.00|1.04)*	1.03 (1.01|1.05)**		1.02 (1.00|1.03)	1.03 (1.01|1.04)*
3 and more		1.07 (1.06|1.09)***	1.09 (1.07|1.12)***		1.07 (1.05|1.09)***	1.09 (1.07|1.12)***
**Communication with father**						
easy	Ref.	Ref.	Ref.	Ref.	Ref.	Ref.
difficult	0.11 (0.09|0.12)***	1.11 (1.09|1.12)***	1.15 (1.11|1.18)***	0.09 (0.08|0.11)***	1.09 (1.07|1.11)***	1.14 (1.10|1.17)***
ACE*Communication with father			*			**
1–2 ACE*difficult			0.96 (0.92|1.00)*			0.96 (0.92|1.00)*
3 ≥ ACE*difficult			0.95 (0.91|0.99)*			0.94 (0.90|0.97)**

*ns* not significant, *p < .05 **p < .01 ***p < .001

The effect of coefficients is multiplicative

### Associations of communication with mother and father with emotional problems and behavioural problems

We assessed the association of communication with mother and with father with emotional problems and behavioural problems (Model 1). We found significant associations between adolescents’ communication with parents (mother and father) and both emotional and behavioural problems. Difficult communication or a complete lack of communication due to the absence of mother and of father increased the probability of emotional and also of behavioural problems.

### Associations of the number of ACE and of communication with mother and father with emotional problems and behavioural problems

We assessed the associations of the number of ACE and of communication with mother and with father with emotional problems and behavioural problems (Model 2). We found both ACE and adolescents’ communication with parents (mother and father) to be significantly associated with both emotional and behavioural problems. More ACE and difficult communication or a complete lack of communication due to the absence of mother and of father increased the probability of emotional and also of behavioural problems (Model 2).

### Moderation of the associations between ACE and EBP by communication with parents (mother and father)

In Model 3, we assessed the interaction of ACE and communication with mother and with father regarding both emotional and behavioural problems. We found a statistically significant interaction of communication with father for the associations of ACE with emotional and behavioural problems. The odds ratio could be interpreted as meaning that having experienced ACE and difficult communication, or a complete lack of communication due to the absence of father, decreased the probability of emotional and even more of behavioural problems when compared to the multiplicative model. We did not find a statistically significant interaction of ACE with communication with mother on either emotional or behavioural problems, showing that for the mother the associations of ACE with EBP were fully multiplicative (Model 3).

## Discussion

The present study shows that EBP among adolescents are more likely in case of difficult communication with parents. We also found an interaction, i.e. that communication with the father moderated the association of ACE with EBP.

We found that difficult communication with both parents is related to emotional and to behavioural problems in adolescents, thus adding to the already existing evidence on overall importance of communication with parents [18–22]. Likewise, recent studies show that negative family interactions between parent and child are associated with higher levels of anxiety and depression [23, 38–40], whereas communication between the parents and the child may be influenced by several factors, such as a divorce of the parents [41, 42]. Moreover, recent studies exploring father-child relationships have proposed an “activation relationship”, where fathers also tend to encourage children to take risks, while at the same time ensuring their safety and security [43–47]. Family communication plays an important role in the occurrence of EBP in adolescence [41]. Difficult communication with mother and father during childhood and adolescence can have a deleterious effect on mental and physical health and on a healthy development, and our study shows that this also holds true for EBP.

Moreover, we found that adolescents’ communication with father moderates the association of ACE with both emotional and behavioural problems among adolescents, but not the communication with mother. This shows that for communication with the mother, the effect of the combination of difficult communication and ACE is multiplicative, i.e. that the strength of the association of ACE with problems is multiplied by the presence of communication problems. However, for communication with the father this is true to a lesser degree. The OR for the interaction of communication with the father with ACE was lower than 1. This indicates that the joint effect of having both ACE and a difficult communication with father was less than multiplicative, i.e. hardly greater than the joint separate effects of having only ACE and of having a difficult communication with father. Such a difference in the role of communication with mother and with father may reflect perceived differences for the adolescent in the nature of the communication. Adolescents report that they talk more and prefer communication with their mothers than their fathers [48, 49], which may result from the fact that the relationship between mother and child is characterized more than father–child interactions by warmth, responsiveness and intimate exchanges [50]. However, previous studies have reported mostly on the different character of communication, whereas our study reveals that those differences result in higher risks for EBP in the case that difficulties or lack of communication, especially with mother, is combined with ACE. We presume that other factors may be involved here, which are linked to more ACE and/or difficult communication with parents and which are associated with a higher risk of more ACE. Adolescents with ACE and difficult communication with their parents might be more likely to get into the care system. In the care system, adolescents with ACE can be provided more specialised care (from e.g. psychologist, social worker), which may have a mitigating effect on the relationship between ACE, communication with parents and EBP.

### Strengths and limitations

The main strengths of our study are its large nationally representative sample and its use of the well-established HBSC methodology. However, this study has also some limitations. First, we used self-reported questionnaires, which can be sensitive for adolescents. However, confidentiality as well as privacy were provided by the self-administration of the questionnaires in the absence of teachers, which prevented potential bias due to data collection. Moreover, previous research has shown the validity of self-reported measurement of EBP as well as ACE [51, 52]. A second limitation is that HBSC study is cross-sectional, which make it impossible to formulate conclusive statements about causality. A third limitation of our study is that we could not discriminate the two categories ‘difficult communication’ and ‘don’t see or have this person’; because the number of respondents in the category ‘don’t see or have this person’ was too small. For future research, it would be appropriate to monitor the impact of these two categories separately.

### Implications

Difficult communication with mother and father was related to EBP among adolescents. We also found that communication with father moderates the association of ACE with both emotional and behavioural problems among adolescents, but not the communication with mother. For communication with mother, the effect of the combination of difficult communication and ACE is multiplicative. However, for father this holds only to a lesser degree. Therefore, special attention should be paid to the adolescents facing the situation of difficult communication with mother and more ACE. This implies that improvement in communication between a parent and child could help decrease the probability of EBP in adolescents with ACE.

Regarding future research, we in particular need longitudinal studies to assess pathways and existing mechanisms on the associations of ACE, communication with parents and EBPs. Finally, further research is needed to examine other factors that enter into the relationship between ACE, communication with parents and EBP, e.g. care providers (psychologist, social worker). Investigating other factors (e.g. care provided) that enter into relationship between ACE, communication with parents and EBP can bring a clearer view of adolescents with EBP and may help to decrease the probability of EBP among adolescents.

## Conclusion

Difficult communication with mother and father is related to EBP among adolescents, and adolescents’ communication with father moderates the association of ACE with both emotional and behavioural problems among adolescents.

## Data Availability

The datasets used and/or analysed during the current study are available from the corresponding author on reasonable request.

## Abbreviations

ACE: Adverse childhood experiences
EBP: Emotional and behavioural problems
FAS: Family affluence
